# Ceftriaxone Treatment for Neuronal Deficits: A Histological and MEMRI Study in a Rat Model of Dementia with Lewy Bodies

**DOI:** 10.1155/2018/4618716

**Published:** 2018-08-01

**Authors:** Ying-Jui Ho, Jun-Cheng Weng, Chih-Li Lin, Mei-Shiuan Shen, Hsin-Hua Li, Wen-Chieh Liao, Nu-Man Tsai, Ching-Sui Hung, Te-Jen Lai, I-Yen Lee

**Affiliations:** ^1^Department of Psychology, Chung Shan Medical University Hospital, Chung Shan Medical University, Taichung 402, Taiwan; ^2^Department of Medical Imaging and Radiological Sciences, Chang Gung University, Taoyuan 33302, Taiwan; ^3^Department of Psychiatry, Chang Gung Memorial Hospital, Chiayi 613, Taiwan; ^4^Institute of Medicine, Chung Shan Medical University, Taichung 402, Taiwan; ^5^Department of Anatomy, Faculty of Medicine, Chung Shan Medical University, Chung Shan Medical University Hospital, Taichung 402, Taiwan; ^6^Department of Pediatrics, Chung Shan Medical University Hospital, Taichung 402, Taiwan; ^7^School of Medical Laboratory and Biotechnology, Chung Shan Medical University, Taichung 402, Taiwan; ^8^Occupational Safety and Health Office, Taipei City Hospital, Taipei 10341, Taiwan; ^9^Department of Psychiatry, Chung Shan Medical University Hospital, Chung Shan Medical University, Taichung 402, Taiwan; ^10^Institute of Medicine, Chung Shan Medical University, Taichung 402, Taiwan; ^11^Division of Urology, Department of Surgery, Tungs' Taichung Metroharbor Hospital, Taichung 43503, Taiwan

## Abstract

Dementia with Lewy bodies (DLB) is characterized by neuronal deficits and *α*-synuclein inclusions in the brain. Ceftriaxone (CEF), a *β*-lactam antibiotic, has been suggested as a therapeutic agent in several neurodegenerative disorders for its abilities to counteract glutamate-mediated toxicity and to block *α*-synuclein polymerization. By using manganese-enhanced magnetic resonance imaging (MEMRI) and immunohistochemistry, we measured the effects of CEF on neuronal activity and *α*-synuclein accumulation in the brain in a DLB rat model. The data showed that CEF corrected neuronal density and activity in the hippocampal CA1 area, suppressed hyperactivity in the subthalamic nucleus, and reduced *α*-synuclein accumulation, indicating that CEF is a potential agent in the treatment of DLB.

## 1. Introduction

Dementia with Lewy bodies (DLB) is a common neurodegenerative dementia, accounting for approximately 10%–25% of the dementia population. DLB patients show progressive cognitive decline and motor dysfunction. Psychiatric symptoms are reported in 99.2% of patients with DLB. DLB patients show higher subscores in the items of delusions, hallucinations, agitation, anxiety, irritation, and aberrant motor behavior, compared to the patients with Parkinson's disease dementia, which seriously affects their quality of life and social activities [1]. There is currently no rat model suitable for searching for more effective medications. In addition to the symptoms of progressive dementia and parkinsonism, accumulation of Lewy bodies in the central nervous system is the hallmark of DLB [2, 3]. Lewy bodies are cytoplasmic eosinophilic protein inclusions of *α*-synuclein (*α*-syn) [4–6]. *β*-Amyloid (A*β*) suppresses the clearance of *α*-syn [7, 8] and accelerates oligomerization of *α*-syn and its toxicity, leading to the deterioration caused by *α*-syn [9]. Moreover, direct or indirect interactions of *α*-syn and A*β* promote their mutual aggregation and accumulation, disturb the function of mitochondria, cause excessive glutamate release in the synapse, and eventually result in excitotoxicity and cell death in the brain [8]. That is, A*β* aggravates the neurotoxicity of *α*-syn in the brain [10].

Viral vectors carrying the *SNCA* gene can constantly express *α*-syn in the host. Some studies have injected these vectors into the hippocampus, cortex, and striatum of rats to model the pathological changes seen in DLB [9, 11]. The advantages of using viral vectors are low cost, fast model establishment, and high expression of genes. Additionally, specific regions of the brain that are of research interest may be targeted [12]. Although viral vectors can deliver specific genes to the brain tissues of the host, the use of the method to transfer genes to larger brain regions, such as the cortex, remains a challenge [13, 14]. In the present study, the *α*-syn gene vector was injected into the lateral ventricle of rats, which diffused with the flow of the cerebrospinal fluid, facilitated *α*-syn expression throughout the regions of the brain, and contributed to the establishment of a rat model for DLB.

There is currently no specific medicine for treating DLB. Because of the cognitive dysfunction in patients with DLB, memantine, an antagonist of N-methyl-D-aspartate (NMDA) receptors, is used to alleviate this symptom. This indicates the involvement of glutamatergic hyperactivity in the disease. Rothstein and colleagues found that ceftriaxone (CEF) increases the expression of glutamate transporter-1 (GLT-1), which can remove excessive glutamate released in the synaptic cleft, reduce excitotoxicity, and yield neuroprotective effects [15]. We have demonstrated that CEF treatment prevents the decrease of neuronal activity [16] and loss of neuronal density in the nigrostriatal system and in the hippocampus in a Parkinson's disease (PD) rat model [17, 18]. Moreover, an *in vitro* model of PD revealed that CEF binds with high affinity to *α*-syn and blocks its polymerization [19].

Manganese-enhanced magnetic resonance imaging (MEMRI) is a valuable imaging tool for directly measuring activity-dependent neuronal events in a living animal [20]. Manganese ion (Mn^2+^) may enter neurons through voltage-gated calcium (Ca^2+^) channels [21] where it is retained there with a biological half-life of 51–74 days according to a study of adult rats' brains [22]. Before MR imaging, the animal was injected intraperitoneally (i.p.) with MnCl_2_, and its neurons took up Mn^2+^ when activated. A quantitative analysis indicated that Mn^2+^ accumulates in neurons according to their activity, because Ca^2+^ elevation is related to the firing activity in excitatory neurons [23]. In addition, its paramagnetic feature enables Mn^2+^ to shorten the longitudinal relaxation time (T1) of water protons and thus enhance the T1-weighted MR signal that is specific to the tissues where Mn^2+^ has accumulated [24], thus rendering it an excellent MRI-detectable contrast agent [25]. Therefore, tissue contrast in Mn^2+^-induced T1 signal intensity in MEMRI may be contingent upon the differential accumulation of Mn^2+^ in active and silent brain regions in which topography can be absolutely quantified by measuring the absolute T1 (or R1 = T1^−1^) value [23, 26].

When combined with pharmacological manipulation, MEMRI can be used to detect changes in neuronal activities that are produced by the treatment. However, whether changes of neuronal activity are accompanied by a loss of neurons in the DLB rat model remains unclear. The purpose of this study was to elucidate the effects of CEF (100 mg/kg/day) on neuronal density and activity in the brain of the DLB rat model using immunohistochemistry and MEMRI.

## 2. Materials and Methods

### 2.1. Animals

Twelve-week-old male Wistar rats (420 ± 30 g; *n* = 25; BioLASCO Taiwan Co Ltd., ROC) were randomly assigned to groups of 3 or 4 and housed in acrylic cages (35 × 56 × 19 cm^3^) in a temperature-controlled animal room (21–25°C) with free access to food and water. Photoperiods in rodent rooms were controlled using an automatic timer set to provide 12 h of light (from 7:00 to 19:00) and 12 h of dark (from 19:00 to 7:00). To minimize defensive behaviors and stress response to the experimenter, prior to the start of the experiment, all animals were handled for 5 min/day on 2 consecutive days. All experimental procedures were performed according to the NIH Guide for the Care and Use of Laboratory Animals and were approved by the Animal Care Committee of Chung Shan Medical University (IACUC approval number 1455). All efforts were made to minimize animal suffering and to reduce the number of animals used [27].

### 2.2. General Procedures

For inducing DLB in the rat model, rats were randomly divided into three groups and underwent stereotaxic brain surgery on day 0 as described in our previous reports [8, 27]. Briefly, the rats were anesthetized by an i.p. injection of Zoletil (20 mg/kg; Virbac, Carros, France) and mounted in a stereotaxic frame. To overexpress *α*-syn, recombinant adeno-associated viral (rAAV) vector containing human *α*-syn gene, *SNCA*, (10 *μ*g/10 *μ*L/rat) was injected into the left lateral cerebral ventricle using the following coordinates adapted from the rat brain atlas: AP: −0.8 mm, ML: −1.5 mm, and DV: −3.6 mm from the bregma, midline, and skull surface, respectively. The A*β*_1–42_ solution (5 *μ*g/2.5 *μ*L/side × 2 sides/rat) was bilaterally infused in the prefrontal cortex using the following stereotaxic coordinates: AP: 1.6 mm, ML: ±2.0 mm, and DV: −2 mm from the bregma, midline, and skull surface, respectively. During the 5 days after surgery, the rats were housed individually in plastic cages and 10% sucrose solution was provided ad libitum to prevent weight loss after surgery and reduce mortality.

Starting from the next day after the surgery (day 1), the animals received the following treatments: the “sham + saline” group (*n* = 5) and “DLB + saline” group (*n* = 7) were injected with saline (1 mL/kg/day, i.p.), and the “DLB + CEF” group (*n* = 6) was injected with ceftriaxone (CEF) (100 mg/kg/day, i.p.) (Roche, Kaiseraugst, Switzerland) for 27 days.

Hydrated manganese chloride (MnCl_2_·4H_2_O, Sigma Aldrich, UK) was dissolved in saline at a concentration of 100 *μ*mol/mL (20 mg/mL). On day 26, all rats received two 1 mL/kg i.p. injections of MnCl_2_ solution separated by 1 h (total dose 40 mg/kg) [28]. Twenty-four hours later (day 27), when Mn^2+^-induced signal enhancement reached a stable asymptotic level [28], the rats were transported to the MR center for MR imaging, anesthetized during imaging, and transported back to the animal room.

On day 28, rats were euthanized by exposure to CO_2_, transcardially perfused with phosphate-buffered saline (PBS) followed by 4% paraformaldehyde in PBS. The brain was then immediately removed and postfixed in PBS containing 30% sucrose and 4% paraformaldehyde at 4°C until use.

### 2.3. MEMRI Data Acquisition

Brain images were acquired on a 7T MRI system (Bruker BioSpec, Karlsruhe, Germany), as described in our previous paper [16]. Briefly, rats were initially anesthetized with 5% isoflurane vapor in oxygen at a flow rate of 500 mL/min. T2-weighted contrast of anatomical images was acquired using the turbo rapid acquisition with relaxation enhancement sequence. T1-weighted images were acquired using a multislice spin-echo sequence. To improve detection sensitivity over the full extent of Mn^2+^ concentrations, R1 images were acquired using a rapid acquisition with relaxation enhancement with variable time of repetition. Six sets of images corresponding to six TRs (ranging from 500 to 3500 ms) were taken during the recovery of the longitudinal magnetization to perform R1 mapping. The R1 maps shown in the paper are representations of the distribution of Mn^2+^ uptake in the region of interest (ROI) that were manually defined in the hippocampus and subthalamic nucleus (STN) in the coronal images. The mean signal intensity for all voxels in the ROIs, averaged for both hemispheres, was used to compare R1 differences between the groups.

### 2.4. Histological Assessment

For histological assessment, frozen coronal sections of the brain (25 *μ*m thick) were cut and mounted on gelatinized slides and maintained in PBS until staining. The regions seen in the stained brain sections were identified according to the rat brain atlas [29] and used for image analysis to measure histological changes, as described previously [17, 28, 30–34]. The ROI was defined in the hippocampus (bregma −2.76 mm to −4.20 mm).

Nissl staining, which is used to identify pyramidal cells in the hippocampus, was performed as described in our previous reports [16, 17, 32]. *α*-Syn staining was used to evaluate the density of *α*-syn immune-positive cells in the brain sections [8, 35]. The coronal sections were rinsed (3 × 5 min each) with 0.05 M Tris buffer (TB), incubated for 20 min at room temperature with 0.3% hydrogen peroxide to block endogenous peroxidase activity, and blocked by incubation for 1 h at room temperature in 100% normal goat serum dissolved in TB containing 10% Triton-100. They were then incubated overnight at 4°C with polyclonal rabbit anti-*α*-syn antibody (1 : 100; GeneTex, USA) and incubated sequentially for 2 h at room temperature with HRP-conjugated goat anti-rabbit IgG (1 : 200; GeneTex, USA), followed by 5 min at room temperature with 3,3′-diaminobenzidine tetrachloride (DAB; Sigma, USA) before being dehydrated in ethanol and xylene. The stained sections of images were acquired using a microscope (ZEISS AXioskop 2, Germany). The density of neurons and *α*-syn-positive cells (number/mm^2^) in the brain sections were counted using the Image Pro Plus Software 6.0 (Media Cybernetics, CA, USA) as described in our previous paper [16].

### 2.5. Data Analysis

SPSS 17.0 statistical software was used for data analysis. Analysis of variance (ANOVA) and the least-significant difference (LSD) post hoc test were used to analyze the data. All results are expressed as the mean ± standard error of the mean (SEM). The level of significance was defined as *P* < 0.05.

## 3. Results

### 3.1. Image Analysis

**Figure 1:**
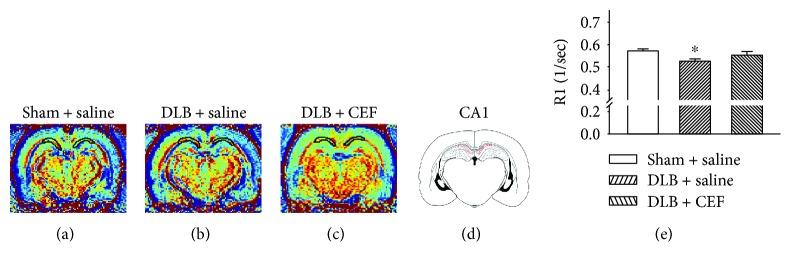
Effect of treatment with ceftriaxone (CEF) on neuronal activity in the hippocampal CA1 area of the DLB rat model. The rats were bilaterally infused with A*β*_1–42_ in the prefrontal cortex and unilaterally infused with viral vectors with the *SNCA* gene in the left lateral ventricle to model DLB in rats. The sham + saline group and DLB + saline group were injected with saline (1 mL/kg/day, i.p.), and the DLB + CEF group was injected with CEF (100 mg/kg/day, i.p.) for 27 days. (a–c) Coronal R1 maps of the rat brain. The regions of interest used for quantitative analysis of Mn^2+^-induced signal enhancement in the hippocampal CA1 area are indicated by the outlines on the schematic (d). (e) Quantitative results. Data are expressed as the mean ± SEM. ^∗^*P* < 0.05, compared with the sham + saline group.

**Figure 2:**
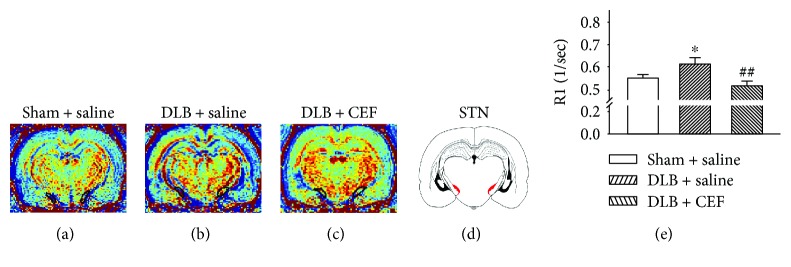
Effect of treatment with ceftriaxone (CEF) on neuronal activity in the subthalamic nucleus (STN) in the DLB rat model. The group names are the same as those in Figure 1. (a–c) Coronal R1 maps of the rat brain. The regions of interest used for quantitative analysis of Mn^2+^-induced signal enhancement in the STN are indicated by the outlines on the schematic (d). (e) Quantitative results. Data are expressed as the mean ± SEM. ^∗^*P* < 0.05, compared with the sham + saline group; ^##^*P* < 0.01, compared with the DLB + saline group.

One-way ANOVA revealed significant differences in brain MRI images between the groups. Compared with the sham + saline group, the DLB + saline group showed decreased R1, Mn^2+^ uptake level in the hippocampal CA1 area (*F*(2,17) = 3.79, *P* = 0.05) (LSD post hoc test, *P* value = 0.03). However, normal Mn^2+^ uptake in the hippocampal CA1 area was seen after CEF treatment because no difference between the sham + saline and DLB + CEF groups was found (Figure 1). One-way ANOVA revealed significant differences in STN MRI images between the groups (F(2,17) = 5.57, *P* = 0.016). The LSD post hoc test revealed a higher Mn^2+^ uptake and neuronal activity in the DLB + saline group, compared with the sham + saline group (*P* < 0.05). The rats receiving CEF treatment showed the same neural activity in the STN as did the controls (Figure 2).

### 3.2. Histological Assay

#### 3.2.1. Density of Pyramidal Neurons in the Hippocampal CA1

**Figure 3:**
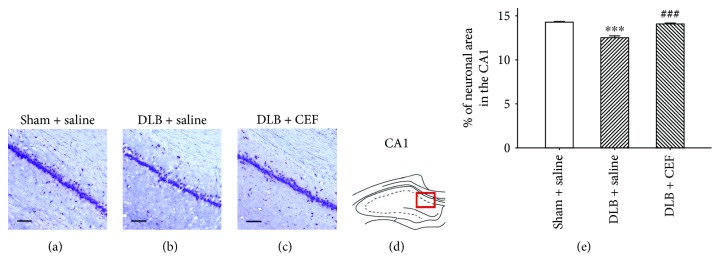
Effect of ceftriaxone (CEF) on the neuronal density in the hippocampal CA1 area of DLB rats. The treatment and group names are the same as those in Figure 1. (a–c) Coronal brain sections of the hippocampal CA1 area; pyramidal cells are revealed through Nissl staining. Magnification, 200x; bar, 100 *μ*m. (d) CA1 region of the hippocampus. (e) Quantitative results. Data are expressed as mean ± SEM. ^∗∗∗^*P* < 0.001, compared with the sham + saline group; ^###^*P* < 0.001, compared with the DLB + saline group.

The ANOVA revealed that density of pyramidal neurons in the hippocampal CA1 showed a significant difference (*F*(2,14) = 56.69, *P* < 0.001), and post hoc analysis showed that rats in the DLB + saline group had a significantly lower neuronal density than did those in the sham + saline group (*P* < 0.001). The DLB + CEF group showed a higher neuronal density than the DLB + saline group (*P* < 0.001) (Figure 3).

#### 3.2.2. Density of *α*-Syn in the Dentate Gyrus

**Figure 4:**
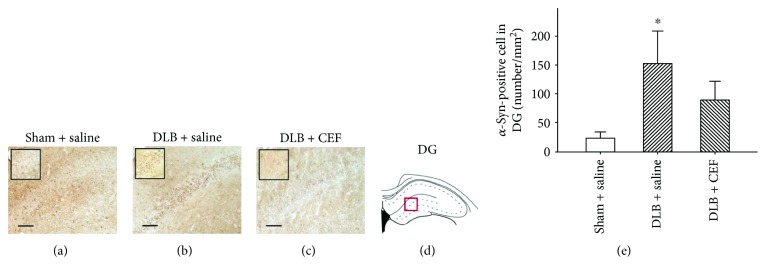
Effect of ceftriaxone (CEF) on the density of *α*-synuclein- (*α*-syn-) positive cells in the DG of DLB rats. The treatment and group names are the same as those in Figure 1. (a–c) Coronal sections in the DG. *α*-Syn-positive cells are indicated by anti-*α*-syn labeling in representative coronal sections. Magnification, 200x; bar, 100 *μ*m. High magnification images (1000x) of *α*-syn-positive cells are shown in the insets. (d) The DG of the hippocampus. (e) Quantitative results. Data are expressed as the mean ± SEM. ^∗^*P* < 0.05, compared with the sham + saline group.

The ANOVA revealed significant differences in the density of *α*-syn-positive cells in the hippocampal dentate gyrus (DG) (*F*(2,14) = 3.91, *P* < 0.05) (partial eta squared = 0.638), and post hoc analysis showed that rats in the DLB + saline group (152 ± 56 number/mm^2^) had a significantly higher density of *α*-syn-positive cells compared with the sham + saline group (23 ± 10 number/mm^2^) (*P* < 0.05). The DLB + CEF group (90 ± 32 number/mm^2^) showed no difference in the density of *α*-syn-positive cells compared with the sham + saline group (Figure 4).

## 4. Discussion

In the DLB rat model, we found an accumulation of *α*-syn in the hippocampal DG and lowered neuronal density and activity in the hippocampal CA1 of the DLB + saline group compared with the sham + saline group. In addition, hyperactivity in the STN was also observed in DLB + saline rats. Treatment with CEF at a dose of 100 mg/kg corrected these neuronal deficits. CEF was previously found to increase GLT-1 expression, resulting in sequestration of excess synaptic glutamate and protection of hippocampal neurons from excitotoxicity [36]. It has been reported that treatment with CEF prevented neurodegeneration in the hippocampus and nigrostriatal system, increasing neuronal activity [16] and improving cognitive function in an MPTP-induced PD rat model [17, 18]. We have previously reported that CEF, at the dosages of 100 and 200 mg/kg, per se did not affect motor function, cognitive behavior, and neuronal density in the hippocampus in healthy rats [17]. These results suggest that CEF can prevent DLB-related neuronal deficits in the brain.

The present study revealed that the injection of A*β* and viral vectors with the *SNCA* gene into the brain caused an accumulation of *α*-syn, neuronal loss, and lowered neuronal activity in the hippocampus, suggesting that this method can induce a useful model of DLB. The pathophysiology of DLB is related to the aggregation of Lewy bodies and Lewy neurites that are formed by *α*-syn accumulation [37, 38], resulting in neurotoxicity and cell loss in the limbic system, brainstem, and cortical regions [5, 39, 40]. Although *α*-syn accumulation is the core pathological feature of DLB, the deposition of extracellular A*β* is also observed in the brains of up to 80% of DLB patients [7]. Direct and indirect interaction between *α*-syn and A*β* has been reported to promote *α*-syn aggregation [8]. A*β* increases the toxicity of *α*-syn [8] and aggravates the relevant defects triggered by *α*-syn, causing neurodegeneration [41]. Thus, in addition to injecting the *α*-syn gene vector into the rat's lateral ventricle, A*β* was also injected into the bilateral prefrontal cortex to more accurately model the pathological characteristics of DLB. In this study, the *α*-syn gene vector was injected into the lateral ventricle of rats, which diffused with the flow of the cerebrospinal fluid and facilitated the expression of *α*-syn throughout the regions of the brain. A high level of *α*-syn accumulation was observed in the hippocampal DG area of DLB + saline rats, which may be involved in lower density of pyramidal neurons in the hippocampal CA1 because the abnormal accumulation of *α*-syn in the limbic system leads to cell loss and dysfunction in the hippocampus [42]. Reportedly, CEF binds to *α*-syn [19] and may thus block polymerization of *α*-syn and exert neuroprotective effects *in vitro* [19], which may underlie the effects of CEF on reducing *α*-syn accumulation and restoring neuronal density and activity in the hippocampus of DLB rats. The subject number in the study is relatively small. The level of *α*-syn in the DG of the hippocampus in the DLB + CEF group may be only partially reversed. To increase statistical power and to provide convincing treatment effect, further study using a larger number of subject is needed.

A*β* oligomers have been reported to play a pivotal role in synaptic impairment and neuronal degeneration [43]. Based on this amyloid hypothesis, the formation of soluble species of A*β* can directly interfere with memory formation and cause synaptic degradation, excitotoxicity, and cell death [44], leading to cognitive decline. Glutamate is one of the main excitatory neurotransmitters in the central nervous system, is involved in synaptic plasticity, memory, and learning, and plays a substantial role in the pathogenesis from the early stages of neurodegenerative diseases. To bind A*β* peptides, NMDA receptors have attracted considerable interest because of their toxic effects and involvement in neurodegeneration [45]. A*β* oligomers interfere with glutamatergic transmission. Under pathological conditions, elevation of A*β* levels blocks glutamate uptake at the synaptic cleft, leading to increased glutamate levels, stimulation of NMDA receptors [46], and activation of extrasynaptic NR2B-enriched NMDA receptors [45], causing an increase of intracellular calcium levels and the activation of metabolic pathways responsible for neuronal shrinkage, synaptic loss, and neurodegeneration [47]. Moreover, A*β* oligomers form complexes with alpha7-nicotinic receptors at presynaptic sites, causing increased levels of glutamate release, and they are involved in synaptic plasticity [48]. These factors indicate that A*β* is responsible for impairments of synaptic plasticity, cell loss, and neuronal death.

Glutamatergic hyperactivity can cause neurodegeneration [49, 50] and may thus be involved in the parkinsonism of DLB. STN neurons express glutamate, innervate the substantia nigra (SNc), and are hyperactivated in PD. Modulation of STN activity by deep brain stimulation improves parkinsonism, indicating that STN hyperactivity is associated with the development of parkinsonian features [51, 52]. STN hyperactivation increases glutamate release, causing excitotoxicity of dopaminergic neurons in the SNc [53]. In the present MEMRI-based study, we observed hyperactivity in the STN in the DLB + saline rats and found that this change was prevented with CEF treatment. This was consistent with our previous findings that CEF prevents dopaminergic and hippocampal degeneration in a PD rat model [17, 18]. We therefore suggest that CEF treatment reduced STN hyperactivity by increasing GLT-1 expression and glutamate reuptake and thus blocked neurodegeneration in the DLB rat model.

The risk of developing dementia in patients with DLB may increase with hippocampal cell loss, because the decline in cognitive function is associated with hippocampal degeneration [54]. Since that hippocampal CA1 is reported to be involved in working memory and cognition [55] and that neurogenesis is observed in the DG [56], dysfunction of the hippocampus may result in impairments of memory and recognition [57, 58]. The hippocampus shows vulnerability of excitotoxic damage due to rich of glutamatergic synapses [36]; excessive glutamate release and excitotoxicity-induced neurodegeneration in this region may lead to memory and recognition impairment, as seen in patients with DLB. The present study revealed hippocampal lesions in the CA1 of DLB rats, which was accompanied by lowered neuronal activity, as measured using MEMRI. Our another study found impairments in cognitive behaviors, for example, object recognition and avoidance learning, in the DLB rats, where deficits were improved after CEF treatment. (Data have been submitted for publication.) Regarding the clinical features of patients with DLB, in addition to cognitive dysfunction, motor impairment is also observed. However, in the experiment, the DLB rats did not exhibit motor dysfunction (data not shown). According to the “1-year rule,” the consensus guidelines to distinguish DLB and PD dementia [59], if cognitive impairment occurs within 1 year following the onset of motor impairment or if cognitive impairment occurs earlier than motor impairment, DLB is diagnosed. Lack of motor impairment in the present study may be due to the short experimental period (only 28 days). The present histological findings showing increased *α*-syn-positive cells in the hippocampus of DLB rats are in line with that *α*-syn pathologies observed in the hippocampus of patients with DLB, which is associated with memory impairment [60]. Furthermore, increasing evidence supports that *α*-syn aggregates may be the real culprit, causing deficits in neurotransmission and neurogenesis in the hippocampus, which is considered to be involved in mechanisms for the hippocampal dysfunctions and associated neuropsychiatric manifestations in synucleinopathy [61]. CEF treatment prevented decreased neuronal density and activity in the area, indicating that CEF may have the potential for treating neurodegeneration and dementia in patients with DLB [17].

Increase of GLT-1 expression may be involved in the CEF-induced prevention of the loss of neuronal function in DLB rats. Excessive release of glutamate and hyperactivity of the glutamatergic system play a critical role in neuronal and behavioral symptoms in neurodegenerative diseases [62, 63]. GLT-1 is responsible for glutamate clearance [64–67] and thus prevents glutamate excitotoxicity [68–70]. In previous studies [17, 18], we found that CEF treatment caused a dose-dependent increase in GLT-1 expression in the brain, had a neuroprotective effect in the hippocampus and nigrostriatal dopaminergic system, and improved cognitive function. We thus suggest that upregulation of GLT-1 and reduction of excitotoxicity may underlie the neuronal protective effect of CEF. Further studies are needed to elucidate the downstream molecular pathway of CEF that modulates glutamate transmission and their neuroprotective effects.

MEMRI data associated with Mn^2+^ uptake are used to quantitatively evaluate neural activity, where R1 value represents the accumulation of Mn^2+^ in neurons [23]. Consistently, our data showed that MEMRI R1 values in the hippocampus reflect cell loss in the DLB rats and treatment effects of CEF against the disease [16]. Higher neuronal activities in the STN of the DLB + saline group indicated similar clinical and pathological characteristics associated with parkinsonism and neurodegeneration [71]. Elevation of STN activities (i.e., a burst pattern of neuronal firing) is also observed in patients with PD [72]. Our previous study found a significant positive correlation between MEMRI R1 and neuronal density in the hippocampus [16], indicating that R1 values in the hippocampus may serve as an indicator of neuronal density of the hippocampus in the DLB rat model. Although the present data revealed hippocampal lesions with abnormal neuronal activity 4 weeks after the surgery, it would be of interest to measure the changes long after the surgery, which would provide evidence to elucidate whether the DLB rat model shares the same progressive features as in patients with DLB.

In conclusion, the results demonstrated that CEF treatment prevented the loss of neuronal density and activity in the hippocampus and also normalized the hyperactivity of the STN in the DLB rat model. Treatment with CEF prevented the accumulation of *α*-syn in the hippocampus. Moreover, the MEMRI R1 value may serve as a suitable indicator for DLB severity and treatment effect. Our data provide support for the use of CEF as a potential therapeutic agent to prevent dementia in patients with DLB.
